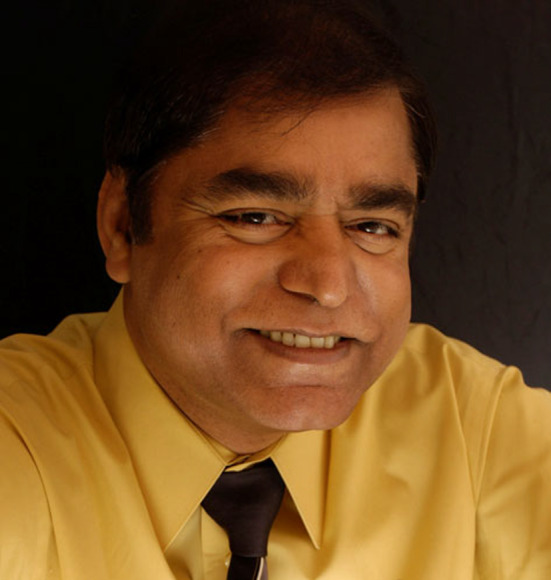# PBJ is now a leading open access plant journal

**DOI:** 10.1111/pbi.12687

**Published:** 2017-01-23

**Authors:** Henry Daniell

**Affiliations:** ^1^ Director of Translational Research University of Pennsylvania Philadelphia PA USA

Welcome to the first issue of the fifteenth volume of *Plant Biotechnology Journal*. I would like to start this editorial by announcing the successful transition of *PBJ* from a subscription‐based journal to an open access journal supported exclusively by authors. This resulted in enhanced free global access to all readers. I applaud the *PBJ* management team for offering free open access to all articles published in this journal in the past 14 years. As the first among the top ten open access plant science journals, based on 2016 citations, *PBJ* is very likely to be ranked among the top three journals publishing original research. *PBJ* is now compatible with mobile platforms, tablets, iPads, and iPhones and offers several new options to evaluate short‐ and long‐term impact of published articles, including Altmetric scores, article readership, and citations.

I want to thank the current editors and editorial staff for their continued commitment to high‐quality science, ethical standards, and for helping to lead *PBJ* in achieving its very high standing among the plant science and biotechnology journals. With the dedication of a highly committed team of Editors and excellent contributions from our authors and critical evaluation by our reviewers, *PBJ* has moved up two notches in ISI ranking (11th of 209) among plant science and (13th of 161) biotechnology/applied microbiology journals in 2015. The journal continues to advance in all key categories including immediacy index, cited half‐life, and article influence score. This success is reflected in an increased interest for the journal, culminating in a 176% increase in the submission of manuscripts between 2012 and 2015, while sustaining a healthy submission rate in 2016, from most geographical areas around the globe. *PBJ* more than doubled the number of external reviewers between 2012 and 2016, and we are thankful for their thoughtful and critical reviews. Doubling the number of reviewers did not increase the average turnaround time (15 days), rewarding our authors with timely decisions on their submissions. Considering *PBJ* is still a very young journal, these are impressive accomplishments.

I am firmly committed to increasing the impact of *PBJ*, to further improve its standing among academic/industry researchers and regulatory agencies, and to increase awareness of this research among the public. While *PBJ* currently publishes full‐length articles encompassing in depth studies, I realized the need for brief timely reports of new inventions. Therefore, I introduced ‘PBJ letters’ for publication of three printed page articles with <1500 words, ten citations, and one illustration.

PBJ Altmetric scores of high impact articles are as high or higher than those published in Science or Nature journals. To further engage the public or policymakers in scientific discussions, PBJ is requesting authors to provide Twitter titles at the time of manuscript submission. In addition, authors are encouraged to share news releases on their articles with the PBJ editorial office so that they are included in Wiley Plant Science tweets @PlantSciNews, which has ~9000 followers.

*PBJ* will continue to publish special issues covering topics of current interest to our readership. *PBJ* published special issues on editing plant genomes (edited by Joseph Petolino, Vibha Srivastava, Henry Daniell) and a focus issue on plant genomes (edited by David Edwards, Rajeev Varshney). In 2017, we anticipate publishing special issues on proteomics and synthetic biology.

In addition to special issues, *PBJ* will continue to publish high‐quality articles in its established areas of strength, as well as expanding into emerging new areas. The number of Associate Editors and Editorial Board members continues to increase to represent several new areas and different continents, not previously represented. We welcome new associate editors Dr Martin Parry (Lancaster University) and Nicola Patron (Norwich Research Park). I convey my sincere thanks for the excellent service offered by all our Associate Editors, especially editors of special issues, new Editorial staff (Jim Ruddock, Samantha Crisp at Oxford) and the Production team (Julie Ann Suliguin, Production Editor, Manila).

I encourage all readers to visit the journal homepage (http://onlinelibrary.wiley.com/journal/10.1111/%28ISSN%291467-7652) to take advantage of open access, keep up to date with the latest developments and to sign up for our automated e‐alerts in order to receive emailed notifications when new issues or Early View articles are published. Please note that readers should ‘opt‐in’ to receive e‐alerts, by visiting the journal homepage and registering at the ‘Get Content Alerts’ area.

Finally, with these and other exciting changes, I firmly believe that *PBJ* will continue to serve the plant research community and offer a platform to publish exciting research findings and new developments.